# New Brilliant Blue G Derivative as Pharmacological Tool in Retinal Surgery

**DOI:** 10.3389/fphar.2020.00708

**Published:** 2020-05-25

**Authors:** Angelo Spadaro, Marco Rao, Miriam Lorenti, Mario Rosario Romano, Antonio Augello, Chiara Maria Eandi, Chiara Bianca Maria Platania, Filippo Drago, Claudio Bucolo

**Affiliations:** ^1^Department of Drug Sciences, University of Catania, Catania, Italy; ^2^Biomedical Sciences, Humanitas University, Milano, Italy; ^3^Section of Hygiene and Food of Animal Origin (SIAOA - UFCM), Department of Veterinary Prevention, Azienda Sanitaria Provinciale (ASP - CT), Catania, Italy; ^4^Department of Ophthalmology, University of Lausanne, Hôpital Ophtalmique Jules-Gonin, Lausanne, Switzerland; ^5^Department of Surgical Science, University of Torino, Torino, Italy; ^6^Department of Biomedical and Biotechnological Sciences, School of Medicine, University of Catania, Catania, Italy; ^7^Center for Research in Ocular Pharmacology-CERFO, University of Catania, Catania, Italy

**Keywords:** inner limiting membrane, retina, vitreoretinal surgery, Brilliant Blue G, pucker, drug design

## Abstract

Our study was aimed at assessing the retinal binding of a new synthetic Brilliant Blue G (BBG) derivative (pure benzyl-Brilliant Blue G; PBB) ophthalmic formulation, to improve vitreoretinal surgery procedure. Protein affinity of the new molecule was evaluated *in vitro* (cell-free assay) and *in silico*. Furthermore, an *ex vivo* model of vitreoretinal surgery was developed by using porcine eyes to assess the pharmacological profile of PBB, compared to commercial formulations based on BBG and methyl-BBG (Me-BBG). PBB showed a higher affinity for proteins (p < 0.05), compared to BBG and Me-BBG. *In vitro* and *in silico* studies demonstrated that the high selectivity of PBB could be related to high lipophilicity and binding affinity to fibronectin, the main component of the retinal internal limiting membrane (ILM). The PBB staining capabilities were evaluated in porcine eyes in comparison with BBG and Me-BBG. Forty microliters of each formulation were slowly placed over the retinal surface and removed after 30 s. After that, ILM peeling was carried out, and the retina collected. BBG, Me-BBG, and PBB quantification in ILM and retina tissues was carried out by HPLC analysis. PBB levels in the ILM were significantly (p < 0.05) higher compared to BBG and Me-BBG formulations. On the contrary, PBB showed a much lower (p < 0.05) distribution in retina (52 ng/mg tissue) compared to BBG and Me-BBG, in particular PBB levels were significantly (p < 0.05) lower. Therefore, the new synthetic Brilliant Blue derivative (PBB) showed a great ILM selectivity in comparison to underneath retinal layers. In conclusion, these findings had high translational impact with a tangible improving in *ex vivo* model of retinal surgery, suggesting a future use during surgical practice.

## Introduction

**Figure 1:**
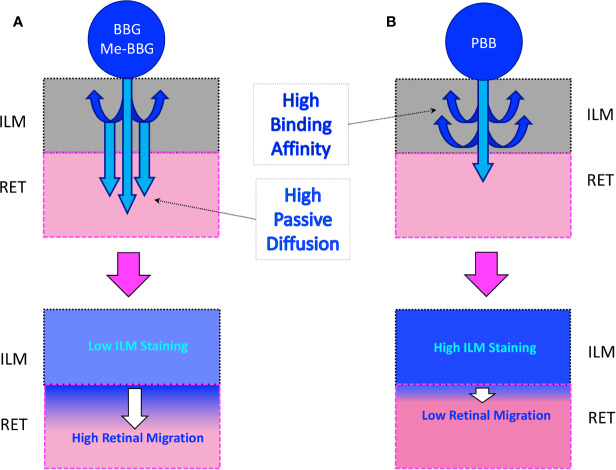
Passive diffusion of molecules from the inner limiting membrane (ILM) to the retina (RET). **(A)** Diffusion of a molecule with low affinity to ILM proteins. **(B)** Diffusion of a molecule with high affinity to ILM proteins.

Retinal surgery includes complex techniques aimed at resolving a variety of conditions, such as macular hole and pucker (also known as epiretinal membrane), a disease that affects the central retina (called macula). The use of vital dyes to stain the retina during ocular surgery is necessary for visualization and removal of thin and transparent membranes, such as the internal limiting membrane (ILM). The mechanism of action of vital dyes rest on binding and staining of proteins, without killing cells (Delaey et al., 2000; Desmettre et al., 2000). Specifically, in vitreoretinal surgery, vital dyes bind and stain ILM proteins, such as fibronectin, a component of extracellular matrix (Rodrigues et al., 2009). The ILM is the boundary between the vitreous body and the retina, and includes the feet of Müller cells and astrocytes (Heegaard et al., 1988). In the central retina, the ILM thickness is around 400 nm; and ILM is characterized by a fibrillar meshwork of different type of collagen fibers surrounded mainly by hyaluronic acid (Heegaard et al., 1988). ILM is a basement membrane that acts as a double-sided sticky tape interface between the vitreous and retina, assisting also the alignment of the Müller cells to the optical axis of the eye (Balasubramani et al., 2010). ILM functions appear non-necessary in the adult eye, and ILM peeling is generally carried out without relevant post-surgery complications (Uechi et al., 2014). The ILM removal has been introduced as adjuvant surgical procedure to improve anatomic and functional outcomes during surgery for macular hole (Lois et al., 2011) and epiretinal membrane (Schechet et al., 2017). Vital dyes for the ILM staining are commonly used in vitreoretinal surgery as ultrashort-acting pharmacological tools, that are used once or more times during the surgery, and then removed few seconds after administration (Januschowski et al., 2018). Despite the widespread use of these molecules in retinal surgery, unmet medical needs still remain unsolved, such as selectivity for ILM and safety. Therefore, surgeons are looking for new molecules capable to selectively stain the ILM, with low affinity (low extend of absorption) to adherent retinal layers, in order to avoid potential cytotoxicity on off-target tissues (**Figure 1**). Protein binding of Brilliant Blue G (BBG), a dye widely used in vitreoretinal surgery (Kawahara et al., 2007; Remy et al., 2008) instead of green indocyanine (potentially toxic to retina), was reported to be mainly guided by hydrophobic interactions (Georgiou et al., 2008). Therefore, we aimed at designing and synthetizing new BBG derivatives, with high lipophilicity in order to improve ILM protein binding affinity, and to avoid diffusion into retinal layers adherent to ILM. We further tested the ILM protein affinity of the new pure benzyl-BBG (PBB^®^) in comparison with BBG and methyl-BBG (Me-BBG), already in the market. Particularly, *in vitro*, *in silico*, and *ex vivo* experimental approaches were aimed to investigate the pharmacological profile of these molecules.

## Methods

### Synthesis

**Figure 2:**
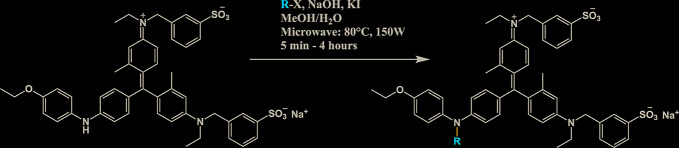
Synthesis scheme of new Brilliant Blue G (BBG) derivatives.

All chemicals used for the synthesis (reagent grade) and chromatographic analysis (HPLC grade) were obtained from Sigma-Aldrich/Merck (Milan, Italy). The synthesis of BBG derivatives (alkyl- and benzyl-BBG series, **Table 1**, **Figure 2**) was carried out as previously reported in the patent (David, 2019), by means of microwave-assisted synthesis protocol (**Figure 2**). The microwave-assisted reactions were done using a CEM Discover System microwave synthesizer (CEM, Cologno al Serio, Bergamo, Italy). Briefly, for the synthesis of alkyl-BBG series (**Table 1**, entry 2–6) the reaction mixture was stirred and irradiated in the microwave synthesizer, and the following conditions were kept constant during 30 min reaction step: 80°C, 150 W, using as solvent MeOH/H_2_O mixture (1/1 v/v). Reaction steps for alkyl series of BBG derivatives were within 5–7 steps. After each step, the reaction vessel was cooled to room temperature and appropriate aliquots of alkylating agent and NaOH were added to the reaction mixture. The overall yields of the aliphatic-N-alkyl derivatives of BBG were within 70–95%. The synthesis of benzylic BBG derivatives (**Table 1**, entry 7–12) was completed within 5–15 min. Briefly, 0.04 mmol of BBG, 1.2 mmol of benzyl-bromide, 1.2 mmol of NaOH, and catalytic amount of KI were added to 4 ml of 1/1 v/v methanol/water mixture. Then, the mixture was irradiated with (150 W) microwaves at 80°C. The overall synthesis yields of the N-benzyl derivatives of BBG were within 80–95%. Alkyl- and benzylic-BBG compounds were purified with flash-chromatography on silica gel, by using a CH_2_Cl_2_/MeOH gradient. Purity of compounds was within 96–99% as determined by HPLC in our lab (data not shown).

**Table 1:** List of designed and synthetized molecules bearing different substituents in the diphenyl nitrogen of Brilliant Blue G (BBG) *pH = 7.4.

Entry	—R	LogD*	MW	Formula
**1**	**—**H	0.50	854.02	C_47_H_48_N_3_NaO_7_S_2_
**2**	**—**CH_3_	0.57	868.05	C_48_H_50_N_3_NaO_7_S_2_
**3**	**—**CH_2_-CH_3_	1.08	882.08	C_49_H_52_N_3_NaO_7_S_2_
**4**	**—**CH_2_-CH_2_-CH_3_	1.58	896.11	C_50_H_54_N_3_NaO_7_S_2_
**5**	**—**CH_2_-CH_2_-OH	−0.77	898.08	C_49_H_52_N_3_NaO_8_S_2_
**6**	**—**CH_2_-CH_2_-OCH_3_	0.53	912.10	C_50_H_54_N_3_NaO_8_S_2_
**7**	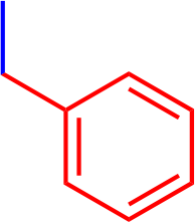	2.2	944.15	C_54_H_54_N_3_NaO_7_S_2_
**8**	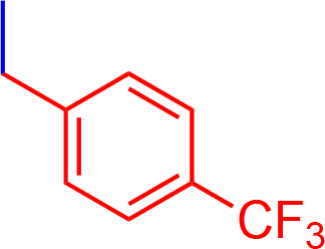	3.05	1012.15	C_55_H_53_F_3_N_3_NaO_7_S_2_
**9**	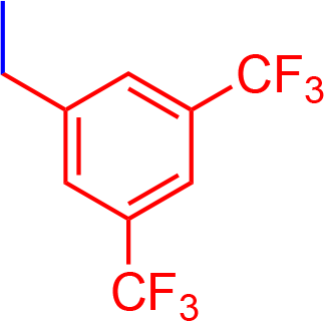	3.55	1080.15	C_56_H_52_F_6_N_3_NaO_7_S_2_
**10**	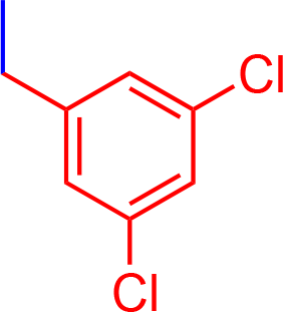	3.50	1013.03	C_54_H_52_Cl_2_N_3_NaO_7_S_2_
**11**	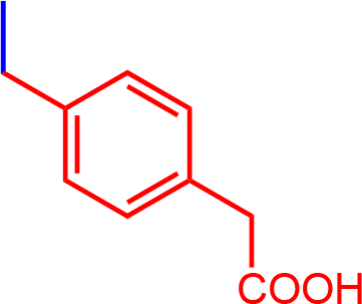	0.36	1002.19	C_56_H_56_N_3_NaO_9_S_2_
**12**	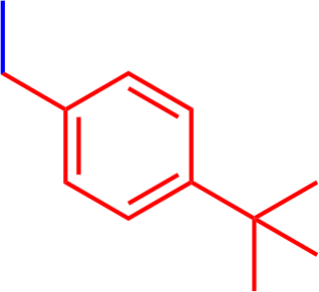	3.90	1000.26	C_58_H_62_N_3_NaO_7_S_2_

### Protein Affinity

In order to have the same dye/protein molar ratio for dye protein affinity assay, a set of molecular weight standards was obtained by mixing 430 nmol of the following proteins: i. fibronectin proteolytic fragment 45 kDa; ii. *E. coli* beta-galactosidase 116.3 kDa; iii. bovine serum albumin 66.5 kDa; iv. chicken ovalbumin 42.7 kDa; v. equine cytochrome C 12.3 kDa. Polyacrylamide gel electrophoresis was carried out loading the molecular weight standard in a 9% precast gels (Biorad), as triplicates on separate lanes. Gel electrophoresis was carried out at 20V for 60–90 min in denaturing conditions. After runs, each lane was cut and soaked overnight in a different staining solution, consisting of dyes dissolved at 0.025% in gel fixative solution (40% methanol, 20% acetic acid). Thereafter, gel lanes were decolored in the fixative solution without dyes, until the background stain reached the minimum level compared to stained protein bands. Images were collected with a scanner at 300 dpi, 16M colors. Densitometric analysis was done using the software ImageJ (Rueden et al., 2017). LogD values (pH 7.4) were calculated *in silico* using ACD/Labs software (version 11.1 2008, ACD Labs). In order to correlate experimental protein affinity of tested vital dyes with predicted binding affinity for fibronectin, one of the main components of ILM, we carried out molecular docking and molecular mechanics generalized born surface area (MM-GBSA) calculations on vital dyes/fibronectin domain complexes. For this purpose, we retrieved from Protein Data Bank repository (https://www.rcsb.org/) the structure of the gelatin binding domain (GBD) of fibronectin (PDB: 3M7P) (Graille et al., 2010). The structure of vital dyes was obtained with the online SMILES translator and structure file generator of NIH Cancer Institute (https://cactus.nci.nih.gov/translate/). The monomer of fibronectin PDB was subjected to the protein preparation task of Schrödinger^®^ maestro. Ionization state of residues was assigned at pH 7.4 with Epik^®^, and then protein was subjected to energy minimization protocol. Sitemap^®^ was used for identification of binding pockets of GBD fibronectin monomer. Docking grid was centered on the biggest pocket of GBD, then, docking of vital dyes on GBD was carried out with the standard precision (SP) docking (Schrödinger^®^) performed with Glide (Schrödinger^®^). The ΔG_binding_ of vital dyes to fibronectin GBD was predicted with MM-GBSA calculation; for this purpose, the VSGB 2.0 solvation model was used and all residues within 15 Å from the ligand were capable to move during application of the minimization energy gradient. Prediction of BBG derivatives ADME properties was carried out with the webserver SwissADME (http://www.swissadme.ch/).

### Ex Vivo Study

*Ex vivo* model of vitreoretinal surgery has been previously reported for evaluation of indocyanine green ILM staining properties (Nakamura et al., 2003). We carried out on swine eyes the same procedure previously reported on primates, with slight modifications. The following formulations have been tested in the *ex vivo* model of vitreoretinal surgery: Brilliant Peel™ (0.025% Brilliant Blue G BBG; Fluoron, Ulm, Germany); ILM-Blue™ (0.025% Brilliant Blue G; Dutch Ophthalmic Research Center (International) B.V, Agrate Brianza, Italy); View-ILM^®^ (0.03% methyl-Brilliant Blue G; Alchimia, Ponte San Nicolò—Padova, Italy); PBB^®^ (0.05% Pure benzyl-Brilliant Blue G; Alfa-Instruments, Casoria, Italy). Along with primates, the swine retina represents the closest resemblance with the human one. Post-mortem swine eyes were obtained from a local slaughterhouse (Department of Veterinary Prevention, Section of Hygiene and Food of Animal Origin, Catania, Italy) by vet supervision (Dr. Antonio Augello); since the eyes were enucleated post-mortem an ethical review process was not required for the present study. Immediately after collection, the eye was placed in a large tube containing BSS solution and transported to the lab. The eye was placed on a glass holder and a 360-degree limbal incision was carried out in order to remove cornea, aqueous, iris ciliary-body, and lens. The vitreous was gently extracted, under operating microscope, with cotton buds and 23-gauge forceps. After core vitreous removal a posterior vitreous detachment was performed. The globe was filled with 2 ml of BSS, then, 40 µl of each formulation were slowly placed over the retinal surface. After 30 s, formulation along with BSS was removed and the retinal surface was rinsed with fresh BSS. The peeling of ILM was carried out by 23-gauge forceps in order to grasp the membrane, and thereafter, the retina underneath the area of peeled ILM was collected. After tissue collection, samples were weighted and stored at −20°C till analysis. The levels of the dye molecules were quantified by means of HPLC analysis.

### High Performance Liquid Chromatography (HPLC) Analysis

ILM and RET tissue samples obtained as described above were homogenized in 250–500 μl of methanol using a micro tissue grinder. Samples were subjected to sonication, carried out with a Branson Sonifier. After that, the samples were centrifuged at 1,000 g for 10 min and filtered on 0.45 μm syringe filters (0.45-μm Spartan filters, Schleicher and Schuell, Dassel, FRG). The filtrates were transferred into clean test tubes and subjected to a gentle stream of nitrogen till dryness. The dried residue was reconstituted in 100 μl of mobile phase by vortexing for 120 s, filtered again on 0.45 μm filters, and, finally, 10–20 μl aliquots were injected into the chromatographic apparatus (each sample was analyzed in triplicate).

HPLC analyses were performed on an Agilent 1260 Infinity II chromatographic system (Agilent Technologies) equipped with a ChemStation OpenLab software (M8307AA), a quaternary pump (G7111B), a diode array detector (DAD, G7111B), and a thermostated column compartment (G1316A). Chromatographic separations were performed using a Kinetex XB-C18 column (100 Å, 100 mm × 4.60 mm, 2.6 μm, Phenomenex). BBG, Me-BBG, and PBB^®^ were analyzed using three different isocratic ternary mobile phases consisting of (A) acetonitrile, (B) methanol, and (C) triethylamine 0.3% (v/v) in water (pH adjusted to 3.0 with trifluoroacetic acid) with different proportions (A:B:C) as follows: BBG 49:5:46 (v/v/v); Me-BBG 54:5:41 (v/v/v); PBB^®^ 62:5:33 (v/v/v). The flow rate was set at 0.5 ml/min and the column was thermostated at 24°C during the analysis. UV spectra were recorded in the range 200–800 nm and chromatograms were acquired at 613 and 316 nm. The analytical method was validated according to ICH guidelines (ICH Guideline, 2005). Identification of the dye molecules (BBG, Me-BBG, and PBB^®^) was determined by HPLC–DAD analysis by comparing the retention times and the UV spectra of the analyzed samples with those of reference standards. Potential interferences from endogenous matrix constituents of ILM and RET tissues were excluded by analyzing the chromatograms of control tissues (untreated eyes), used for HPLC calibration. Furthermore, peak-purity tests (Gilliard and Ritter, 1997; Stahl, 2003) were used to demonstrate the absence of coeluting peaks. Peak-purity tests were done with OpenLab software (Agilent Technologies) using photodiode-array detector spectra. Eleven-point calibration curves were set up for all standards to test the linearity of the UV-DAD response. Linear regression was performed, using OpenLab software (Agilent Technologies), on the calibration data points of each dye tested to determine slope, intercept, and correlation coefficient (R^2^). Calibration standards were prepared by spiking 10 μl of appropriate working solutions of the respective dye standard into control (untreated eyes) ILM and retinal samples. Calibration standards were processed as reported in the sample preparation procedure and analyzed by HPLC. The limits of detection (LOD) and quantification (LOQ) were determined in biological samples spiked with progressively lower concentrations of the analytes. The LOD and LOQ were established with an S/N ratio of 3:1 and 10:1, respectively (n=6). Precision and accuracy were determined at three concentration levels in ILM and RET within the calibration range by assaying replicates (n = 5) of samples spiked with the three tested dyes (25,800 and 12,800 ng/ml), and processed as the unknowns. To determine intra- and inter-day precision and accuracy, matrix samples were analyzed in replicates on the same day and on three different days, respectively. Intra- and inter-day precision was reported as relative standard deviation (RSD%), with an acceptability limit set at ≤15%. Accuracy was calculated by comparison of mean assay results with the nominal concentrations, with acceptability limits set at ±15%.

### Statistical Analysis

Data are expressed as mean ± SD values, n=8. Statistical analysis was carried out with application of one-way ANOVA, followed by Tukey post-hoc test, for multiple comparison between groups. Differences between groups were considered statistically significant given p-values <0.05. Statistical analysis and graph design were carried out with GraphPad Prism 7.0 (San Diego, CA, USA).

## Results

### Synthesis

Microwave-assisted synthesis is a well-established approach to decrease reaction times and increase yields of several reactions in organic synthesis (Kappe, 2004). In fact, the reaction time for the synthesis of the alkyl-BBG series (**Table 1**, **Figure 2**) by microwave assisted synthesis was within 0.5–4 h, compared to longer reaction time (168–192 h, data not shown) of conventional synthetic approaches. Also for the benzylic BBG derivatives (**Table 1**, **Figure 2**) the designed microwave-assisted synthesis ensured very fast reaction rate within 5–15 min (David, 2019).

### Design of Synthetic Dyes

In a simplified model of dye retinal distribution (**Figure 1**), the key factors are: (i) the passive diffusion process that, according to the Fick’s Law, depends on concentration gradient and diffusion coefficient of the dye; (ii) the dye binding affinity to ILM proteins. The balance between these two factors affects the retinal distribution of the dye. Our goal was to design BBG derivatives with higher binding affinity to ILM proteins, in order to get an ILM trapping of the new dyes, and avoiding dye migration to retinal layers (**Figure 1**, panel B). BBG binds the main proteins of ILM, collagen IV, laminin, and fibronectin (Duhamel, 1983) forming a blue dye-protein complex (Compton and Jones, 1985). BBG binds to protein with a combination of electrostatic and hydrophobic interactions. The heteropolar bonding occur between the sulfonic groups of BBG and basic amino acids of proteins (Lys, His, and Arg) (Tal et al., 1985). Importantly, other studies suggest that hydrophobic forces play a major role in the interactions between BBG and aromatic amino acid protein residues (Phe, Tyr, Trp) (Compton and Jones, 1985; Georgiou et al., 2008; Katrahalli et al., 2010). For these reasons, we synthesized a series of BBG derivatives, by introducing alkyl- or benzyl-moieties on the diphenylic nitrogen of BBG, in order to increase lipophilicity and the number of putative hydrophobic interactions of BBG derivatives with proteins of ILM (Caron et al., 2006). We identified the benzyl-BBG derivative (PBB^®^, **Table 1**, logD=2.20) as the best candidate to be further developed, bearing physical-chemical properties that lead to a balance between lipophilicity and water solubility. Furthermore, the arylic moieties, compared to alkyl-moieties, can ensure increasing non-covalent interactions by means of π-bonds (π stacking, polar-π, cation-π, and anion-π), with aromatic residues of proteins (Ward and Beswick, 2014). The higher lipophilicity of the PBB^®^, combined with a suitable concentration, ensured higher binding affinity to ILM proteins, avoiding relevant dye migration to retinal layers (**Figure 1**, panel B), as demonstrated by the *ex vivo* studies.

### Protein Interaction

**Figure 3:**
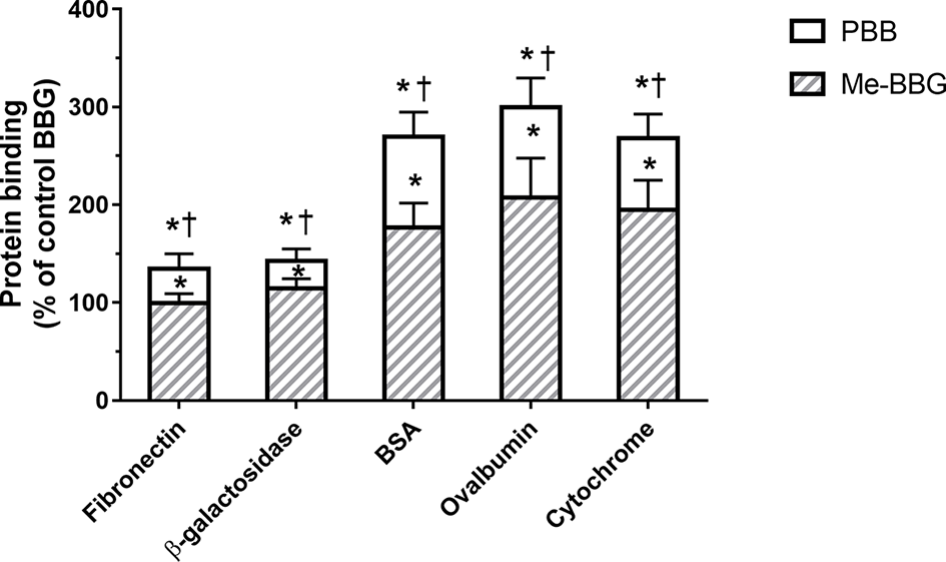
Protein binding [% of control Brilliant Blue G (BBG)]. Pure benzyl-Brilliant Blue G (PBB) binds with higher affinity to proteins, compared to BGG and methyl-Brilliant Blue G (Me-BBG). *p < 0.05 *vs.* BBG; ^†^p < 0.05 *vs.* Me-BBG.

**Figure 4:**
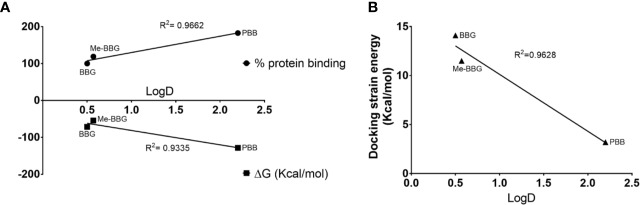
Hydrophobic interactions are the driving forces of dye binding affinity to proteins. **(A)** Upper quadrant LogD vs experimental % dye protein binding (for BBG, the % values were set to 100), bottom quadrant LogD vs predicted dye ΔG_binding_ to fibronectin; **(B)** LogD vs docking strain energy of dyes bound to fibronectin.

**Figure 5:**
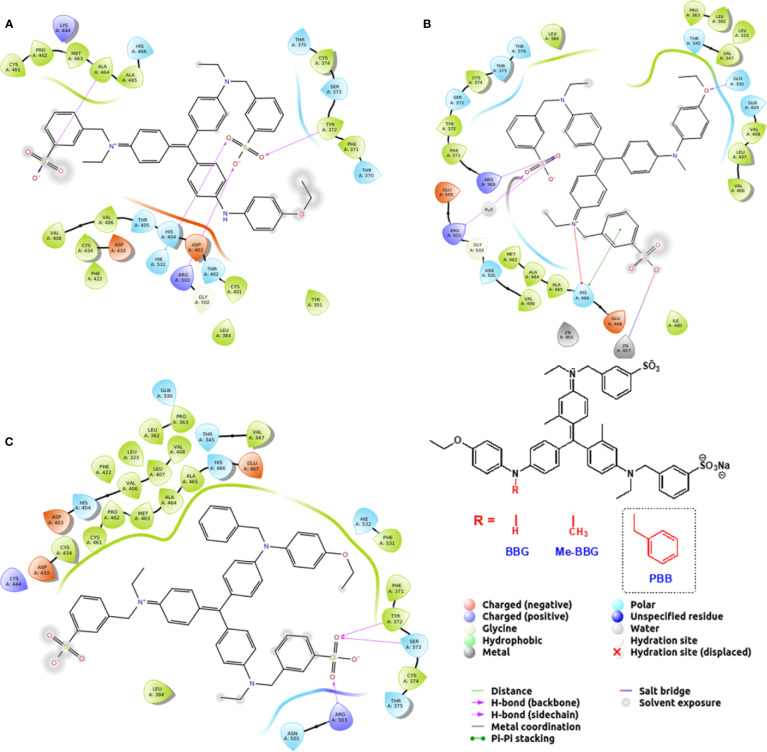
Pure benzyl-Brilliant Blue G (PBB) forms with fibronectin more hydrophobic contacts than Brilliant Blue G (BBG) and methyl-Brilliant Blue G (Me-BBG). **(A)** BBG pose; **(B)** Me-BBG pose; **(C)** PBB pose.

The PBB^®^ binding, quantified as staining intensity, to proteins bands analyzed with SDS-page was significantly higher (p < 0.05), compared to BBG (**Figure 3**). Driving forces of BBG binding to proteins is generally attributed to hydrophobic and Van der Waals interactions, and to some extent also to bonds of sulphonic anion group with basic and polar residues (Georgiou et al., 2008). Particularly, it was also reported that hydrophobic forces play a major role in the interaction between BBG and BSA (Compton and Jones, 1985; Katrahalli et al., 2010). We found that PBB^®^ had higher protein affinity (% staining) compared to BBG and Me-BBG (**Figure 3**). Predicted logD values (pH 7.4) showed that PBB^®^ has higher lipophilicity (LogD = 2.2) compared to BBG (LogD = 0.5) and Me-BBG (LogD = 0.57). Fibronectin, a high molecular weight glycoprotein (400–500 kDa), is the main component of ILM, and its expression in ILM is increased in elderly and diabetic patients (Ljubimov et al., 1996; To et al., 2013). The *in silico* study confirmed that PBB^®^ binding to fibronectin is mainly driven by hydrophobic interactions, in comparison to BBG and Me-BBG. Furthermore, this result fits with *in vitro* data and prediction of LogD. We predicted dye binding affinity for fibronectin, through combination of ligand docking and MM-GBSA calculation. PBB^®^, BBG and Me-BBG were docked to the monomer of GBD of fibronectin (Graille et al., 2010). After docking, complexes were subjected to MM-GBSA calculation in order to predict the affinity of vital dyes to fibronectin. The predicted ΔG_binding_ of PBB^®^ (−128,32 Kcal/mol) to fibronectin GBD was significantly lower (more negative, more favorable) compared to Me-BBG (−54.28 Kcal/mol) and BBG (−71.22 Kcal/mol). Furthermore, the LogD (lipophilicity index) correlated with mean percentage (%) of dye protein binding (**Figure 3**, 100 was set for BBG), predicted ΔG_binding_ and also docking strain energy; although the dataset is limited to three congeneric dyes (**Figure 4**). Furthermore, the predicted receptor docking strain energy (the energy of receptor after binding to compounds) inversely correlated with LogD (**Figure 4**) and increasing steric hindrance of R (**Figure 5**) in BBG (14.1 Kcal/mol), Me-BBG (11.5 Kcal/mol), and PBB^®^ (3.2 Kcal/mol). Moreover, as shown in **Figure 5**, PBB^®^ bound to fibronectin interacts through Van der Waals interactions with more hydrophobic residues (green residues) of fibronectin GBD, compared to BBG and Me-BBG. ADME prediction confirmed that all compounds: i. are poorly soluble; ii. do not cross the BBB barrier because they are all substrates of p-glycoprotein drug efflux pump; and iii. are not metabolized by cytochromes P450 (1A2, 2C19, 2C9, 2D6, 3A4). Due to series of violations of Lipinski rule all the tested BBG derivatives are no drug-like compounds, although BBG is a potent selective non-competitive antagonist of purinergic ionotropic P2X7 receptor (Bartlett et al., 2014).

### HPLC Method Validation

Under the chromatographic conditions reported in the *Methods* section, the retention times of BBG, Me-BBG, and PBB^®^ were 4.68, 4.58, and 4.69, respectively. The correlation coefficients for standards of all dyes included in the matrices ILM and RET (from untreated eyes) were higher than 0.9999 and the y-intercept values were relatively low (**Table 2**). The LOD values in analyzed tissues ILM/RET were 9.85/10.10, 10.10/9.90, and 9.57/9.34, for BBG, Me-BBG, and PBB^®^, respectively. While, the LOQ values in ILM/RET were 29.84/30.29, 30.59–30.00, and 29.01–28.30, for BBG, Me-BBG, and PBB^®^, respectively (**Table 2**). The acceptability criterion recommended by ICH guidelines was reached for all obtained analytical data, with overall intra- and inter-day RSD% not exceeding 6%, and accuracy values within the 95.5–103.5% range (data not shown). These results demonstrated that the used analytical method was reproducible and reliable for biological analyzed samples.

**Table 2:** Calibration data, limits of detection (LOD), and quantification (LOQ) obtained by High Performance Liquid Chromatography (HPLC) analysis.

Matri	Dye	Regression equation	R^2^	Linear range	LOD	LOQ
		y=peak area, x=ng/ml		(ng/ml)	(ng/ml)	(ng/ml)
**ILM**	BBG	y = 0.17014693·x+0.5023796	0.99999	0.7800–12,800	9.85	29.84
	Me-BBG	y = 0.17444827·x+0.5150798	0.99998	0.7800–12,800	10.10	30.59
	PBB^®^	y = 0.16301218·x+0.4812452	0.99999	0.7800–12,800	9.57	29.01
**RET**	BBG	y = 0.17273799·x+0.5100300	0.99998	0.7800–12,800	10.00	30.29
	Me-BBG	y = 0.17102771·x+0.504980	0.99998	0.7800–12,800	9.90	30.00
	PBB^®^	y = 0.16135010·x+0.475048	0.99999	0.7800–12,800	9.34	28.30

BBG, Brilliant Blue G; Me-BBG, methyl-Brilliant Blue G; PBB^®^, pure benzyl-Brilliant Blue G; ILM, inner limiting membrane; RET, retina.

### Porcine Eye Model

**Figure 6:**
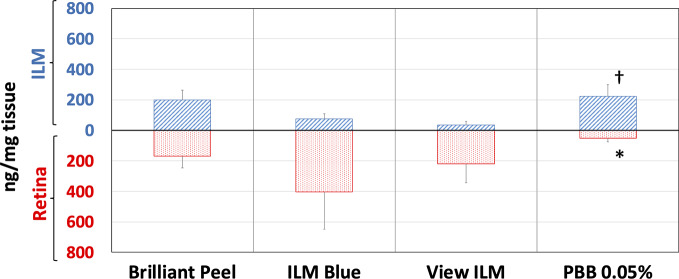
Retinal internal limiting membrane (ILM) and underneath retinal layers dye distribution in porcine eye. Brilliant Peel (Brilliant Blue G), ILM Blue (Brilliant Blue G), View ILM (methyl-Brilliant Blue G), and PBB (Pure benzyl-Brilliant Blue G). *p < 0.05 *vs.* Brilliant Peel, ILM Blue and View ILM; ^†^p < 0.05 *vs.* ILM Blue and View ILM.

The *ex vivo* model of vitreoretinal surgery has strengthened the data of *in vitro* cell free dye protein binding affinity study. Through the *ex vivo* model of vitreoretinal surgery we evaluated the binding of tested dye formulations to ILM and retina (**Figure 6**). Dye quantification was carried out with HPLC in isolated retinal tissues. HPLC analysis confirmed that PBB^®^ was the dye that selectively accumulated with higher concentration in ILM, compared to the retina (RET), after ILM peeling procedure. In fact, PBB^®^ levels, detected in the ILM, were significantly (p < 0.05) higher, with 2.9- and 6.0-fold increase, compared to BBG (Brilliant Peel™, ILM Blue™) and Me-BBG (View ILM^®^) formulations, respectively. PBB^®^ formulation provided higher, even though not statistical significant levels of dye content in ILM compared to Brilliant Peel™ formulation (BBG). It is worthy of note, that the PBB^®^ formulation was mainly selective for ILM staining and did not cross this membrane, in fact, PBB^®^ retinal distribution was significantly lower (p < 0.05) compared to other tested formulations. The administration of the 0.05% PBB^®^ formulation, in the *ex vivo* model of vitreoretinal surgery, provided dye levels of 52 ng/mg in the retina. Compared to PBB^®^, the retinal levels of the other tested formulations ILM Blue™ (BBG), View ILM^®^ (Me-BBG), and Brilliant Peel™ (BBG) were 7.9, 4.3, and 3.3-fold higher, respectively.

## Discussion

In general, the use of vital dyes in vitreoretinal surgery is very common and represents an important tool during the surgical procedure. Vital dyes, for their defined mechanism of action, are not supposed to damage the stained cells, however, in the past, some concerns about potential retinal toxicity has been raised (Röhrig et al., 2019). Awareness about vital dyes safety was mainly related to dye uptake and retention in retinal layers, underneath to the ILM (Cheng et al., 2005). Before the spread of BBG, indocyanine was the most common used molecule in vitreoretinal surgery. Indocyanine toxicity was accounted to non-selective compound uptake into retinal structures underneath the ILM, such as Müller cells (Haritoglou et al., 2002). The potential toxic effects of indocyanine led to development of BBG as new molecule for vitreoretinal surgery. BBG shows a good safety profile *in vitro* and *in vivo* on Müller cells (Kawahara et al., 2007) and retinal ganglion cells, respectively (Remy et al., 2008). In the present study, we designed a BBG derivative with high selectivity for retinal ILM, and poor selectivity for the other retinal structures, underneath the ILM. Retinal binding of the new BBG derivative (PBB^®^) has been assessed by *in vitro*, *in silico*, and *ex vivo* models. Accordingly to this multidisciplinary approach, the present study would have a great translational impact, because the results generated from the bench can be translated to the clinical practice. In order to design new BBG derivatives with high ILM affinity, we synthesized a series of BBG derivatives by introducing alkyl or benzyl moieties at the diphenylic nitrogen of BBG, bearing increasing lipophilicity, without affecting the chromophore and staining properties (**Table 1**, **Figure 2**) (David, 2019). Brilliant Blue G, Me-BBG, and PBB^®^, among all BBG derivatives, were tested to confirm that increased molecule lipophilicity resulted also in greater ILM staining selectivity. An *in vitro* cell free binding affinity assay, an *in silico* study, and an *ex vivo* model of vitreoretinal surgery were used to figure out the pharmacological profile of BBG, Me-BBG, and PBB^®^. Our *in vitro* and *in silico* studies confirmed that BBG derivatives binding affinity for proteins, such as fibronectin, is mainly driven by hydrophobic interactions (Georgiou et al., 2008). The *ex vivo* study confirmed that increased molecule lipophilicity and protein binding/staining lead to increased ILM selectivity of the dye. The ILM is characterized by a well-organized tridimensional network of proteins. Most important proteins that are included in the 3-D scaffold of ILM are type IV collagen, fibronectin, laminin, nidogen-1, and heparan sulfate proteoglycans (agrin, perlecan, and collagen XVIII). In elderly subjects, fibronectin, collagen IV, agrin, and laminin are abundant (Kohno et al., 1987b). In diabetic patients the thickness of ILM is reported to be higher than healthy subjects (To et al., 2013). Furthermore, fibronectin was overexpressed in ILM of diabetic patients, along with tenascin (Kohno et al., 1987a; Russell et al., 1991; Roy et al., 1996; To et al., 2013; Peynshaert et al., 2019). Indeed, fibronectin due to its abundance in ILM of elderly and diabetic patients, along with collagen, represents the main target of vital dyes used in vitreoretinal surgery. Therefore, we assessed the protein affinity of BBG derivatives through SDS-page of standard proteins such as fibronectin, *E. coli* beta-galactosidase, bovine serum albumin, chicken ovalbumin, and equine cytochrome C. PBB^®^ showed the highest affinity, quantified as protein staining intensity after SDS-page electrophoresis (**Figure 3**). We also carried out an *in silico* study, to support the hypothesis that increased dye lipophilicity would increase the protein binding affinity and hydrophobic interactions in the dye-fibronectin complex (**Figures 4** and **5**). Therefore, BBG derivatives binding to fibronectin was predicted through computational studies, i.e. molecular docking and MM-GBSA calculation. For this purpose, we retrieved the structure of the GBD of fibronectin (Graille et al., 2010). The new synthetic dye PBB^®^ showed the higher affinity for proteins used in the SDS-page assay, as well as predicted affinity for GBD of fibronectin, compared to the other tested BBG derivatives. These results are related to the high PBB^®^ hydrophobicity, (**Figure 4**). Furthermore, PBB^®^ binding to proteins, expressed in the ILM, was confirmed by the *ex vivo* model of vitreoretinal surgery. PBB^®^ showed a higher ILM selectivity and a negligible retinal absorption, compared to BBG and Me-BBG (**Figure 6**). In other words, the PBB^®^ ensured a better interaction with ILM proteins, leading to dye entrapment in ILM, with reduced diffusion to other retinal layers (**Figure 1**). The *ex vivo* paradigm set-up in the present study is reliable, cheap, and mimics clinical conditions allowing to evaluate the dye distribution in retinal membranes with high levels of accuracy. The ideal dye for vitreoretinal surgery should be a high grade of purity compound, in order to avoid any potential interference during surgery. It has been reported that retinal damage can occur after treatment with vital dyes, and this is linked to the passive absorption of molecules in the retinal layers underneath the ILM (Kanda et al., 2004; Kawahara et al., 2007; Ueno et al., 2007; Maia et al., 2009; Ejstrup et al., 2012; Savary and Kodjikian, 2014; Almeida et al., 2015; Hurst et al., 2017) (**Figure 1**). On the basis of predicted ADME properties of PBB^®^ the risk of iatrogenic retinal damage is very low, also due to poor absorption into the retina. The new molecule PBB^®^, due to its physical-chemical characteristics and binding properties, is sequestrated by the retinal ILM, with negligible passive diffusion in the underneath retinal layers. In conclusion, these findings had high translational impact suggesting that the new molecule could be useful in retinal surgical practice, even though clinical trials would be warranted in order to assess safety and efficacy profile.

## Data Availability Statement

The raw data supporting the conclusions of this article will be made available by the authors, without undue reservation, to any qualified researcher.

## Author Contributions

Conceptualization: AS and CB. Validation: AS and CB. Data analysis: AS, CP, ML, and MR. Investigation: AS, CP, ML, MR, and CB. Writing—original draft preparation: AS, CP, AA, ML, MR, MRR, and CB. Writing—review and editing: AS, CP, ML, MRR, CE, FD, and CB. Supervision: AS and CB. Funding acquisition: AS and CB.

## Conflict of Interest

The authors declare that the research was conducted in the absence of any commercial or financial relationships that could be construed as a potential conflict of interest.

## References

[B1] Almeida F. P. P. De Lucca A. C. Scott I. U. Jorge R. Messias A. (2015). Accidental subretinal brilliant blue G migration during internal limiting membrane peeling surgery. JAMA Ophthalmol. 133, 85–88. 10.1001/jamaophthalmol.2014.3869 25321324

[B2] Balasubramani M. Schreiber E. M. Candiello J. Balasubramani G. K. Kurtz J. Halfter W. (2010). Molecular interactions in the retinal basement membrane system: A proteomic approach. Matrix Biol. 29, 471–483. 10.1016/j.matbio.2010.04.002 20403434

[B3] Bartlett R. Stokes L. Sluyter R. (2014). The p2x7 receptor channel: Recent developments and the use of p2x7 antagonists in models of disease. Pharmacol. Rev. 66, 638–675. 10.1124/pr.113.008003 24928329

[B4] Caron G. Ermondi G. Scherrer R. A. (2006). Lipophilicity, polarity, and hydrophobicity. Compr. Med. Chem. II. 5, 425–452. 10.1016/b0-08-045044-x/00135-8

[B5] Cheng S. N. Yang T. C. Der Ho J. Hwang J. F. Cheng C. K. (2005). Ocular toxicity of intravitreal indocyanine green. J. Ocular Pharmacol. Ther. 21, 85–93. 10.1089/jop.2005.21.85 15718832

[B6] Compton S. J. Jones C. G. (1985). Mechanism of dye response and interference in the Bradford protein assay. Analyt. Biochem. 151, 369–374. 10.1016/0003-2697(85)90190-3 4096375

[B7] David F. (2019). WO2019068935 - Brilliant Blue (BBG) dye derivatives and staining compositions comprising the same for selectively staining biological substrates.

[B8] Delaey E. Van Laar F. De Vos D. Kamuhabwa A. Jacobs P. De Witte P. (2000). A comparative study of the photosensitizing characteristics of some cyanine dyes. J. Photochem. Photobiol. B.: Biol. 55, 27–36. 10.1016/S1011-1344(00)00021-X 10877064

[B9] Desmettre T. Devoisselle J. M. Mordon S. (2000). Fluorescence properties and metabolic features of indocyanine green (ICG) as related to angiography. Survey Ophthalmol. 45, 15–27. 10.1016/S0039-6257(00)00123-5 10946079

[B10] Duhamel R. C. (1983). Differential Staining of Collagens and Non-Collagens with Coomassie Brilliant Blue G and R. Topics Catalysis. 3, 195–204. 10.1016/S0174-173X(83)80003-X 6191910

[B11] Ejstrup R. La Cour M. Heegaard S. Kiilgaard J. F. (2012). Toxicity profiles of subretinal indocyanine green, Brilliant Blue G, and triamcinolone acetonide: A comparative study. Graefe’s Arch. Clin. Exp. Ophthalmol. 10.1007/s00417-011-1886-3 22173216

[B12] Georgiou C. D. Grintzalis K. Zervoudakis G. Papapostolou I. (2008). Mechanism of Coomassie brilliant blue G-250 binding to proteins: A hydrophobic assay for nanogram quantities of proteins. Analyt. Bioanalyt. Chem. 250, 669–677. 10.1007/s00216-008-1996-x 18327568

[B13] Gilliard J. A. Ritter C. (1997). Use of simulated liquid chromatography-diode array detection data for the definition of a guide curve in peak purity assessment by spectral comparison. J. Chromatography A. 786, 1–11. 10.1016/S0021-9673(97)00552-9

[B14] Graille M. Pagano M. Rose T. Ravaux M. R. van Tilbeurgh H. (2010). Zinc Induces Structural Reorganization of Gelatin Binding Domain from Human Fibronectin and Affects Collagen Binding. Structure. 10.1016/j.str.2010.03.012 20541508

[B15] Guideline I. C. H. (2005). Validation of analytical procedures: text and methodology Q2 (R1). IFPMA: Geneva.

[B16] Haritoglou C. Gandorfer A. Gass C. A. Schaumberger M. Ulbig M. W. Kampik A. (2002). Indocyanine green-assisted peeling of the internal limiting membrane in macular hole surgery affects visual outcome: A clinicopathologic correlation. Am. J. Ophthalmol. 134, 836–841. 10.1016/S0002-9394(02)01816-0 12470751

[B17] Heegaard S. Jensen O. A. Prause J. U. (1988). Structure of the vitread face of the monkey optic disc (Macaca mulatta). SEM on frozen resin-cracked optic nerveheads supplemented by TEM and immunohistochemistry. Graefe’s Arch. Clin. Exp. Ophthalmol. Albrecht Von Graefes Archiv. Fur. Klinische Und Experimentelle Ophthalmol. 226, 377–383. 10.1007/bf02172971 3049259

[B18] Hurst J. Schnichels S. Spitzer M. S. Bartz-Schmidt K. U. Farecki M. L. Szurman P. (2017). Negative Effects of Acid Violet-17 and MBB Dual In Vitro on Different Ocular Cell Lines. Curr. Eye Res. 42, 1209–1214. 10.1080/02713683.2017.1285942 28358247

[B19] Januschowski K. Irigoyen C. Pastor J. C. Srivastava G. K. Romano M. R. Heimann H. (2018). Retinal toxicity of medical devices used during vitreoretinal surgery: A critical overview. Ophthalmologica. 240, 236–243. 10.1159/000488504 30001544

[B20] Kanda S. Uemura A. Yamashita T. Kita H. Yamakiri K. Sakamoto T. (2004). Visual field defects after intravitreous administration of indocyanine green in macular hole surgery. Arch. Ophthalmol. 240, 236–243. 10.1001/archopht.122.10.1447 15477455

[B21] Kappe C. O. (2004). Controlled microwave heating in modern organic synthesis. Angewandte Chem. Int. Ed. 43, 6250-6284. 10.1002/anie.200400655 15558676

[B22] Katrahalli U. Kalanur S. S. Seetharamappa J. (2010). Interaction of bioactive coomassie brilliant blue g with protein: Insights from spectroscopic methods. Sci. Pharm. 78, 869–880. 10.3797/scipharm.1008-15 21179322 PMC3007605

[B23] Kawahara S. Hata Y. Miura M. Kita T. Sengoku A. Nakao S. (2007). Intracellular events in retinal glial cells exposed to ICG and BBG. Invest. Ophthalmol. Visual Sci. 48, 4426–4432. 10.1167/iovs.07-0358 17898261

[B24] Kohno T. Sorgente N. Goodnight R. Ryan S. J. (1987a). Alterations in the distribution of fibronectin and laminin in the diabetic human eye. Invest. Ophthalmol. Visual Sci. 28, 515–521 3557864

[B25] Kohno T. Sorgente N. Ishibashi T. Goodnight R. Ryan S. J. (1987b). Immunofluorescent studies of fibronectin and laminin in the human eye. Invest. Ophthalmol. Visual Sci. 28, 506–514 3549611

[B26] Ljubimov A. V. Burgeson R. E. Butkowski R. J. Couchman J. R. Zardi L. Ninomiya Y. (1996). Basement membrane abnormalities in human eyes with diabetic retinopathy. J. Histochem. Cytochem. 44, 1469–1479. 10.1177/44.12.8985139 8985139

[B27] Lois N. Burr J. Norrie J. Vale L. Cook J. McDonald A. (2011). Internal limiting membrane peeling versus no peeling for idiopathic full-thickness macular hole: A pragmatic randomized controlled trial. Invest. Ophthalmol. Visual Sci. 52, 1586–1592. 10.1167/iovs.10-6287 21051731

[B28] Maia M. Farah M. E. Rodrigues E. B. Malerbi F. K. (2009). Subretinal Brilliant Blue G migration during internal limiting membrane peeling. Br. J. Ophthalmol. 93, 1687. 10.1136/bjo.2008.151597 19939799

[B29] Nakamura T. Murata T. Hisatomi T. Enaida H. Sassa Y. Ueno A. (2003). Ultrastructure of the vitreoretinal interface following the removal of the internal limiting membrane using indocyanine green. Curr. Eye Res. 27, 395–399. 10.1076/ceyr.27.6.395.18189 14704923

[B30] Peynshaert K. Devoldere J. Minnaert A. K. De Smedt S. C. Remaut K. (2019). Morphology and Composition of the Inner Limiting Membrane: Species-Specific Variations and Relevance toward Drug Delivery Research. Curr. Eye Res. 44, 465–475. 10.1080/02713683.2019.1565890 30638413

[B31] Röhrig S. Farecki M. L. Boden K. T. Haus A. Gutfleisch M. Jung S. (2019). Negative Effects Of Vital Dyes After Uneventful Vitreomacular Surgery. Retina (Philadelphia Pa.). 39, 1772–1778. 10.1097/IAE.0000000000002231 29965936

[B32] Remy M. Thaler S. Schumann R. G. May C. A. Fiedorowicz M. Schuettauf F. (2008). An in vivo evaluation of Brilliant Blue G in animals and humans. Br. J. Ophthalmol. 10.1136/bjo.2008.138164 18653608

[B33] Rodrigues E. B. Costa E. F. Penha F. M. Melo G. B. Bottós J. Dib E. (2009). The Use of Vital Dyes in Ocular Surgery. Survey Ophthalmol. 92, 1142–1147. 10.1016/j.survophthal.2009.04.011 19682624

[B34] Roy S. Cagliero E. Lorenzi M. (1996). Fibronectin overexpression in retinal microvessels of patients with diabetes. Invest. Ophthalmol. Visual Sci. 37, 258–266. 8603829

[B35] Rueden C. T. Schindelin J. Hiner M. C. DeZonia B. E. Walter A. E. Arena E. T. (2017). ImageJ2: ImageJ for the next generation of scientific image data. BMC Bioinf. 10.1186/s12859-017-1934-z PMC5708080 29187165

[B36] Russell S. R. Shepherd J. D. Hageman G. S. (1991). Distribution of glycoconjugates in the human retinal internal limiting membrane. in. Invest. Ophthalmol. Visual Sci. 18, 529 2055693

[B37] Savary P. Kodjikian L. (2014). Intraretinal brilliant blue G infiltration during internal limiting membrane peeling. Graefe’s Arch. Clin. Exp. Ophthalmol. 252, 1017–1019. 10.1007/s00417-014-2631-5 24789532

[B38] Schechet S. A. Devience E. Thompson J. T. (2017). The effect of internal limiting membrane peeling on idiopathic epiretinal membrane surgery, with a review of the literature. Retina. 37, 873–880. 10.1097/IAE.0000000000001263 27617536

[B39] Stahl M. (2003). Peak purity analysis in HPLC and CE using diode-array technology Application. Agilent Technol. 5988-8647EN, 1–16.

[B40] Tal M. Silberstein A. Nusser E. (1985). Why does Coomassie Brilliant Blue R interact differently with different proteins? A partial answer. J. Biol. Chem. 260, 9976–9980. 4019521

[B41] To M. Goz A. Camenzind L. Oertle P. Candiello J. Sullivan M. (2013). Diabetes-induced morphological, biomechanical, and compositional changes in ocular basement membranes. Exp. Eye Res. 116, 298–307. 10.1016/j.exer.2013.09.011 24095823

[B42] Uechi G. Sun Z. Schreiber E. M. Halfter W. Balasubramani M. (2014). Proteomic view of basement membranes from human retinal blood vessels, inner limiting membranes, and lens capsules. J. Proteome Res. 13, 3693–3705. 10.1021/pr5002065 24990792

[B43] Ueno A. Hisatomi T. Enaida H. Kagimoto T. Mochizuki Y. Goto Y. (2007). Biocompatibility of brilliant blue G in a rat model of subretinal injection. Retina. 27, 499–504. 10.1097/IAE.0b013e318030a129 17420705

[B44] Ward S. E. Beswick P. (2014). What does the aromatic ring number mean for drug design? Expert Opin. Drug Discov. 9, 995–1003. 10.1517/17460441.2014.932346 24955724

